# CIS7/419: Information Content of Conventional Patient Files in Internal Medicine

**DOI:** 10.2196/jmir.1.suppl1.e12

**Published:** 1999-09-19

**Authors:** C Bobrowski, G Kreymann

**Affiliations:** ^1^Universitäts-Krankenhaus Eppendorf, HamburgGermany

**Keywords:** Hospital Information Systems, Computerized Medical Records, Filing, Information Management, Internal Medicine

## Abstract

**Introduction:**

Migration from conventional patient files to an electronic patient records requires to estimate the amount of information generated per case. This is particularly necessary when planning a distributed environment, i.e. an Intranet. As part of our intranet design, the information content of patient files in internal medicine was measured.

**Methods:**

A random sample of patient files was drawn form the archive of the Medizinische Kernklinik Department of Internal Medicine The sample consisted of the last 75 cases reposited until 12 May, 1999. Length of stay was documented for every case. All sheets of paper were counted and classified into the categories final report, physical examination/patient history, laboratory findings, other technical findings, referrals and other papers. The class of laboratory findings included results from the clinical laboratory, and from genetics and microbiology. Results from technical examinations such as imaging, ECG, function tests and pathology were aggregated into the class of other technical findings. Referrals were defined as in-hospital specialist consultations. Finally, the union of physical examination/patient history, laboratory findings, other technical findings, and referrals was defined as intentionally gathered information (in short: intentional papers), whereas the other papers were defined as routine documentation papers (in short: documentary papers).

**Results:**

Length of stay was a right skewed distribution with 7.66 ± 5.92 days (mean ± sd), median 6 and mode 4. The number of pages per file was also right skewed with 60.6 ± 35.8 pages, median 53 and mode 58. The volume of laboratory reports was between 11 and 40 pages per file in 90.6 % of the cases (11 to 20 pages: 31,1%; 21 to 30 pages: 48,7 %; 31 to 40 pages: 10,8 %). The volume of technical findings was less than 20 pages per file in 85,1 % (6 to 10 pages: 32.4 % ; 11 to 15 pages: 32.4 %). The distribution of referrals was strongly right skewed, with 54.1 % of cases without referrals at all (1 referral: 16.2 %; 2 referrals: 18.9 %; 3 referrals: 5.4 %; 4 or more referrals: 5.3 %)Intentional pages per file were 33.2 ± 24.4, skewness 2.7. Documentary pages per file were 27.4 ± 14.9, skewness 1.2. The total number of pages was correlated to the length of stay with a regression coefficient of r^2 = 0.64. The result for the number of intentional pages vs. length of hospital stay was r^2 = 0.61.

**Discussion:**

The skews of the above distributions provide strong evidence for the assumption that a few documents are retrieved frequently, both during the patient stay, and after discharge, typically at re-admission. Laboratory reports have simple data structures but their volume is high. In contrast, other technical reports may have a complex data structure but their volume is low. As a consequence, information presentation or semi-automatical information aggregation must reflect these differences.

